
JOURNAL INFORMATION
==============================
NLM Title Abbreviation: Atlanta Med Surg J (1884)
NLM Journal ID: 101251623

Atlanta Medical and Surgical Journal (1884)

Publisher: Biomedical Journal Digitization Project

ARTICLE INFORMATION
==============================
PMCID: PMC9201857
Article version: 1
Subjects: News and Notes

News and Notes

Print publication date: 1899 Feb
Volume: 15
Volume Series: New Ser
Issue: 12
First page: 833
Last page: 838
License: This work is free of known copyright restrictions. For more information, please see PMC Back Issue Digitization.
License URL: https://creativecommons.org/publicdomain/mark/1.0/

==============================

### *NEWS AND NOTES.*

---

Dr. R. C. Loyd, of Flat Creek, Ga., paid The Journal a visit during the month.

---

Dr. A. F. Griggs has removed from West Point, Ga., to Atlanta, and located his office in the Prudential building.

---

Dr. W. A. Chisolm, who moved to Waco, Texas, from Atlanta, in 1897, committed suicide, according to the daily papers, on January 16th, from an overdose of strychnine.

---

Dr. Faust, of Denham, S. C., has moved to Flovilla, Ga.

---

Dr. J. T. Longino has been elected mayor of Fairburn, Ga.

---

Dr. B. M. Patterson, of Macon, Ga., died in that city on January 7th, at the age of 65.

---

An epidemic of meningitis is said to have prevailed in and around Albany, Ga., during the month of January.

---

Dr. R. T. Dozier has again been elected as mayor of Sasser, Ga. The Sasser people evidently know a good citizen.

---

A boy, eight years of age, bled to death from a broken tooth in Decatur, Ga., on January 20th. The condition, hemophilia, was hereditary in his family.

WANTED.—By a physician of five years' experience, a location where there is room for a doctor and a chance to make a living. Married. Address box 111, Monroe, Ga.

---

AN evangelist named Pulliam has recently held "religious fervor" meetings in Waycross, Ga., and during these a number of people are said to have gone off in a trance, lasting twenty-four hours. One is said to have been a bride of two days, which, in her case, makes it excusable.

---

SEWANEE MEDICAL COLLEGE, Tennessee, University of the South, graduated last session fifty-five students in medicine and twenty-four in pharmacy.

---

DR. LARKIN SMITH, health officer at Nashville, Tenn., was recently presented with a handsome silver tea service by his associates in the city health office.

---

DR. PAUL PAQUIN has resigned his position as secretary of the State Board of Health of Missouri. He resigned to take charge of a hospital in North Carolina.

---

IT is reported in the daily papers that Mrs. Julia Spence, of Claiborne county, Tennessee, aged 63, has just cut four front teeth. She has been toothless for six years.

---

DR. JNO. GUITERAS, Professor of Pathology in the University of Virginia, has, according to reports, resigned this chair to accept the Chair of Medicine in the University of Havana.

---

THE Talladega Medical Association held its annual session in Talladega, Ala., on January 10th. A most excellent program was rendered. Dr. Jno. T. Dixon was elected president.

THE annual session of the Medical Department of Kentucky University began on January 2d, 1899, under unusually favorable auspices. On December 31st, the following gentlemen, formerly professors in the Kentucky School of Medicine, were elected full professors in Kentucky University of their respective departments: Professor J. B. Marvin, B.S., M.D., LL.D., Professor of Principles and Practice of Medicine and Clinical Medicine; Professor J. M. Holloway, A.M., M.D., Professor of Surgery and Clinical Surgery; Professor C. W. Kelly, C.M., M.D., Professor of Anatomy; Professor S. E. Woody, A.M., M.D., Professor of Chemistry and Diseases of Children. Kentucky University on January 1st, celebrated the 100th anniversary of its foundation; it being originally Transylvania University.

---

IT is officially announced that the New York Cancer Hospital will hereafter be known as the General Memorial Hospital.

---

A NEW medical journal, called the *Medical Dial*, has made its first appearance. It is issued monthly in Minneapolis, Minn.

---

PROFESSOR RÖNTGEN, of X-ray fame, has resigned his appointment at the University of Würzburg to accept the Professorship of Physics in the University of Leipsic.

---

PROF. PHILIP KNOLL, Professor of Experimental Pathology at the German University of Prague, is to succeed to the Chair of Experimental Pathology in the University of Vienna, made vacant by the death of Professor Stricker.

---

THE Sixth International Otological Congress will be held in London, on August 8, 9, 10, 11 and 12, 1899, under the presidency of

Dr. Urban Pritchard, Professor of Otology at King's College, London.

---

A BOARD of officers will be convened at the United States Marine Hospital, Chicago, Ill., February 14th, 1899, for the purpose of examining candidates for admission to the grade of assistant surgeon in the United States Marine Hospital Service.

---

IT has been said that the hospitals of Paris are in an unsanitary condition and lack accommodations sufficient to meet the demands made upon their resources. As a result of recent examination, the superintendent of the charitable institutions of Paris asserts that it would require at least $10,000,000 to put the hospitals of that city in proper condition.

---

A CHARGE of six cents for medicines or for surgical dressings is now being made in the out-patient department of the London Hospital. It is found that those who can afford this nominal charge give it willingly, the sum being remitted in the case of those really unable to pay it. Five thousand dollars a year will be made by the adoption of this plan.

---

THE daily papers report the successive deaths in Metropolis, Ill., of a whole family from pneumonia. Dr. H. Y. Mangum, the head of the family, was first to succumb, and twenty-four hours later his daughter. Three days later his wife also died, and his youngest son was buried the following day, and later the grandmother died. The eldest son alone survives.

---

***NOTICE.—This JOURNAL and THE INTERNATIONAL JOURNAL OF SURGERY one year for $1.00 cash.***

M. JOSEPH has recently observed three cases of "razor chancre," and Rosenthal states that he almost always has one case at least of extragenital chancre in his service. An abrasion of the skin seems to be a necessary preliminary to infection; a razor cut, a crushed finger, burnt mouth or something of the kind. Infection is not necessarily derived from the barber shop, but may follow a kiss on the cut spot later, "although the number of barber's assistants constantly undergoing specific treatment certainly indicates some danger in the shops."—*Derm. Ztft.*, November, 1898.

---

DR. HENRY SCHMEITZER, an expert chemist, has analyzed a quantity of packages of so-called phenacetin; these samples were found to be mixtures of acetanilid with flour, starch, sugar, bicarbonate of soda, chalk and citric acid. Acetanilid costs fifty cents a pound, while phenacetin costs eighty-five cents an ounce. According to the New York Board of Health, other drugs largely adulterated are citrate of magnesia, bisulphate of quinin, cream of tartar and acid phosphates. The inference is that in using these drugs one should make sure that he has procured the genuine article.

---

**NOTICE.—This JOURNAL and THE INTERNATIONAL JOURNAL OF SURGERY one year for $1.00 cash.**

---

SASHES worn by army surgeons, says the *Medical Record,* have a history. A great many people do not know why army surgeons wear green sashes. It is not so much an insignia of rank as it is a protection to the wearer. According to the code of war, surgeons are never shot or taken prisoners. To deliberately shoot a surgeon while he is wearing his sash is considered a violation of the code, punishable by death. Because of this provision, surgeons of one army never refuse to look after the wounded of the other army if it is possible for them to do so. During the civil war it was often the case that after a battle the field hospitals would contain almost an equal number of men dressed in blue and gray. The Federal army had the best surgeons and the best stores, and a wounded Confederate considered himself in great luck if he was removed to a Federal hospital to be cared for by Federal surgeons and physicians.

---

### A VICTIM.

O the grip—
The terrible grip!
I'm wondering still if he'll get me this trip!
If he'll cough me to glory
And finish my story—
The grip—the terrible grip!

O the grip—
The terrible grip!
I'm wondering still if he'll founder the ship!
Or still keep a-body
A-taking his "toddy"—
The grip—the terrible grip!

O the grip!—
(Ain't it time for a "nip?"
Be sure that the hour—the minute don't slip!)
I'm almost as lucky
As folks in Kentucky,—
So, here's to the grip—to the grip!

—F. L. STANTON, in *Atlanta Constitution.*

---

**NOTICE.—This JOURNAL and THE INTERNATIONAL JOURNAL OF SURGERY one year for $1.00 cash.**
